# Long-Term *Helicobacter pylori* Infection Switches Gastric Epithelium Reprogramming towards Cancer Stem Cell-Related Differentiation Program in *Hp*-Activated Gastric Fibroblast-TGFβ Dependent Manner

**DOI:** 10.3390/microorganisms8101519

**Published:** 2020-10-02

**Authors:** Gracjana Krzysiek-Maczka, Aneta Targosz, Urszula Szczyrk, Tomasz Wrobel, Malgorzata Strzalka, Tomasz Brzozowski, Jaroslaw Czyz, Agata Ptak-Belowska

**Affiliations:** 1 Department of Physiology, The Faculty of Medicine, Jagiellonian University Medical College, 31-531 Cracow, Poland; aneta.targosz@uj.edu (A.T.); urszula.szczyrk@uj.edu.pl (U.S.); malgorzata.strzalka@uj.edu.pl (M.S.); agata.ptak-belowska@uj.edu.pl (A.P.-B.); 2 Department of Cell Biology, The Faculty of Biochemistry, Biophysics and Biotechnology, Jagiellonian University, 30-837 Cracow, Poland; t.wrobel@doctoral.uj.edu.pl (T.W.); jarek.czyz@uj.edu.pl (J.C.)

**Keywords:** *Helicobacter pylori*, gastric cancer, activated fibroblasts, cancer associated fibroblasts (CAFs), epithelial–mesenchymal transition (EMT), TGFβ1, cancer stem cells, epithelial–myofibroblast transition

## Abstract

*Helicobacter pylori* (*Hp*)-induced inflammatory reaction leads to a persistent disturbance of gastric mucosa and chronic gastritis evidenced by deregulation of tissue self-renewal and local fibrosis with the crucial role of epithelial–mesenchymal transition (EMT) in this process. As we reported before, *Hp* activated gastric fibroblasts into cells possessing cancer-associated fibroblast properties (CAFs), which secreted factors responsible for EMT process initiation in normal gastric epithelial RGM1 cells. Here, we showed that the long-term incubation of RGM1 cells in the presence of *Hp*-activated gastric fibroblast (*Hp*-AGF) secretome induced their shift towards plastic LGR5^+^/Oct4^high^/Sox-2^high^/c-Myc^high^/Klf4^low^ phenotype (l.t.EMT^+^RGM1 cells), while *Hp*-non-infected gastric fibroblast (GF) secretome prompted a permanent epithelial–myofibroblast transition (EMyoT) of RGM1 cells favoring LGR^−^/Oct4^high^/Sox2^low^/c-Myc^low^/Klf4^high^ phenotype (l.t.EMT^−^RGM1 cells). TGFβ1 rich secretome from *Hp*-reprogrammed fibroblasts prompted phenotypic plasticity and EMT of gastric epithelium, inducing pro-neoplastic expansion of post-EMT cells in the presence of low TGFβR1 and TGFβR2 activity. In turn, TGFβR1 activity along with GF-induced TGFβR2 activation in l.t.EMT^−^RGM1 cells prompted their stromal phenotype. Collectively, our data show that infected and non-infected gastric fibroblast secretome induces alternative differentiation programs in gastric epithelium at least partially dependent on TGFβ signaling. *Hp* infection-activated fibroblasts can switch gastric epithelium microevolution towards cancer stem cell-related differentiation program that can potentially initiate gastric neoplasm.

## 1. Introduction

Gastric cancer (GC) remains the fourth most common cause of cancer-related deaths worldwide. It is estimated that 90% of all stomach tumors are malignant, with gastric adenocarcinoma comprising 95% of the total number of malignancies [1]. Despite the improvement in surgery, chemo-radiotherapy, and targeted therapy, patients still suffer from cancer recurrence and metastasis [2]. These result from the biological properties of tumor cells, including their high invasiveness, rapid proliferation, and the activity of anti-apoptotic systems [3] leading to considerable GC mortality. The asymptomatic gastric infection with the Gram-negative bacterium *Helicobacter pylori* (*Hp*) accounts for the high incidence of cancer recurrence, metastasis, and increased chemoresistance. This WHO-designated class I carcinogen considerably interferes with the curative resection of aggressive GCs [1,4], resulting in the cascade of events related to the interplay between *Hp* infection, host factors, and environmental factors. The *Hp*-induced inflammatory reaction leads to a persistent disturbance of gastric mucosa and chronic gastritis. This is evidenced by deregulation of tissue self-renewal and local fibrosis with the crucial role of epithelial–mesenchymal transition (EMT) in this process [5]. *Hp* induces a plethora of different signal transduction processes that trigger a complex multi-step process leading to inflammation and carcinogenesis [6,7,8]. These pathways are responsible for control of cellular responses such as proliferation, apoptosis, epithelial dedifferentiation, and motility. So, it seems undiscussable that the interplay between different cell types ranging from gastric epithelial cells, glands, immune cells, and stem cells is crucially important for the development and progression of *H. pylori*-associated carcinogenesis [9,10,11]. Besides cells of the innate and adaptive immune system, the tumor microenvironment is to a large degree made up of cancer-associated fibroblasts (CAFs). Fibroblasts might be an important *Hp* target since Necchi [12] identified the presence of *Hp* not only in epithelial cells and intraepithelial intercellular spaces, but also in the underlying *lamina propria* and stromal tumor. Recently, the direct interaction between this bacterial pathogen and fibroblasts has been proposed [13], suggesting that *Hp* interacts with several other cells beyond its direct effect on gastric *epithelium* s, namely with connective tissue components including fibroblasts.

Transforming growth factor-beta1 (TGFβ1) represents a pleiotropic superfamily of cytokines belonging to the class of the most potent EMT inducers. TGFβ1 acts via the activation of a hetero-oligomeric receptor complex consisting of the type II receptor (TGFβRII) and the type I ALK5 receptor (TGFβR1). These complexes activate a range of intracellular signal transduction pathways [14] involved in the regulation of a multitude of biological processes including tissue homeostasis, angiogenesis, cell migration, and differentiation [15,16]. Under physiological conditions, TGFβ has been proposed to inhibit cell proliferation [17]. It also induces EMT-type II-related local fibrosis, which otherwise accompanies the inflammatory responses and tissue repair under normal conditions. Nevertheless, TGFβ1 is also overproduced in many tumors and plays a dual role in GC progression and metastasis [17,18]. These events are associated with the limited benefit of GC treatment regimens via the stimulatory effect on the tumor stroma, in particular on cancer-associated fibroblast formation [18]. CAFs represent an important element of the tumor microenvironment [19,20,21] responsible for EMT-type III initiation in epithelial cell compartments, which is a prerequisite for CAF-induced tumor promotion/progression [22,23,24,25]. Apart from TGFβ1, this process is achieved by cancer-related secretion of a range of other pro-inflammatory and tumor promoting factors [26,27,28].

CAFs can also promote the expansion of cancer stem cells (CSCs) or induce generation of CSCs from differentiated cancer cells [29]. Accumulating evidence reveals an intimate link between embryonic development, stem cells, and cancer formation [30]. Cancer stem cells share features with true stem cells by having the capacity to self-renew in de-differentiated state, to generate heterogeneous types of differentiated progeny, and to give rise to the bulk tumor [31,32]. These activities are related to the pluripotency circuitry, particularly to so-called “Yamanaka factors” such as Oct4, Klf4, Sox2, and c-Myc [30]. 

TGFβ1-induced EMT has been linked with the acquisition of stem-like characteristics by neoplastic cells [33]. At the advanced stages of cancer development, TGFβ1 can accelerate cell proliferation, induce EMT, and enhance the invasion and migration of stem-like cancer cells, resulting in tumor metastasis [17,34]. We recently reported that *Hp* (cagA+vacA+) induces the activation of gastric fibroblasts (*Hp*-AGFs) into the cells possessing CAF phenotype [35]. Here, we concentrated on the long-term effect of *Hp*-activated gastric fibroblasts (*Hp*-AGF) secretome on the pattern of phenotypic reprogramming of gastric epithelial cells. In particular, we addressed the role of *Hp* signaling in the determination of *Hp*-AGF-mediated pluripotency of EMT-committed gastric epithelial cells, with a special emphasis to the role of TGFβ signaling in this process.

## 2. Materials and Methods

### 2.1. Bacterial Hp Strain

The *Hp* strain expressing CagA and VacA cytotoxins (43504 *Hp cagA+vacA+* (*s1*/*m1*)) was purchased from American Type Culture Collection [36,37]. Stock cultures were maintained at −70 °C in Brucella Broth (Becton Dickinson, New Jersey, NJ, USA), supplemented with 10% fetal bovine serum and 10% glycerol. The cultures of bacteria were grown on Columbia Agar with 5% fresh horse blood (BioMerieux, Marcy l’Etoile, France). The plates were incubated under microaerophilic conditions at 37 °C for 3–5 days. Before the co-incubation with fibroblasts, *Hp* strain was suspended in PBS (Sigma-Aldrich, Saint Louis, MO, USA) and immediately transferred to the dishes containing fibroblasts.

### 2.2. Technique of Rat Gastric Fibroblasts Isolation and Their Activation towards Fibroblasts Possessing CAFs Characteristic 

Gastric samples were harvested from 8-week-old Spraque-Dowley rats and extensively washed with sterile PBS to remove contaminating debris. Primary and secondary fibroblast culture was established as described previously [36]. The cells were cultured in DMEM containing 10% FBS and antibiotics (penicillin, streptomycin, and amphotericin B; Sigma A5955, Sigma-Aldrich, Saint Louis, MO, USA). The flasks were maintained in a humidified atmosphere of 5% CO_2_ at 37 °C, and the medium was changed every 2 days [36]. Before the co-incubation with fibroblasts, *Hp* strain was suspended in PBS, counted with Densimat Densitometer (bioMerieux, Marcy l’Etoile, France), and immediately transferred to the dishes containing fibroblasts. Then, 0.25 × 10^6^ fibroblasts were infected with 1 × 10^9^ of live *Hp* and incubated in humidified atmosphere for 72 h (*Hp*-AGFs). As for the control, gastric fibroblasts were cultured for 72 h in DMEM + 10% FBS (GF) [22,35,36,37].

### 2.3. Isolation of Hp-AGF and Normal Gastric Fibroblast (GF)-Conditioned Media

Gastric fibroblasts were co-cultured with *Hp* (cagA+vacA+) strain for 72 h [35]. Then, the *Hp* was washed out from fibroblasts and the medium was changed into DMEM + 10% FBS and antibiotics (penicillin, streptomycin, and amphotericin B; Sigma A5955, Marcy l’Etoile, France). The culture dishes were maintained in a humidified atmosphere of 5% CO_2_ at 37 °C for 4 h, and then the incubation fluid was again replaced with fresh portion of the medium. Fibroblasts were then left in fresh medium for 96 h. After 96 h, the supernatant was collected (abbreviated in this paper as *Hp*-AGF supernatant). The same procedure was applied to the control, normal gastric fibroblast culture (herein abbreviated as GF). To confirm the absence of viable bacteria, the *Hp*-AGF supernatants were applied on the plates with Columbia Agar with 5% fresh horse blood (BioMerieux, Marcy l’Etoile, France). The plates were incubated under microaerophilic conditions at 37 °C for up to 7 days. No visible signs of bacterial growth were detected. Additionally, the *Hp*-AGF secretome-target epithelial cells were checked for *Hp* 16S rRNA expression. All supernatants were stored in 4 °C up to two weeks.

### 2.4. Long-Term Influence of Supernatants Collected from Hp-AGFs and GFs on Epithelial RGM1 Cells

**Scheme 1:**
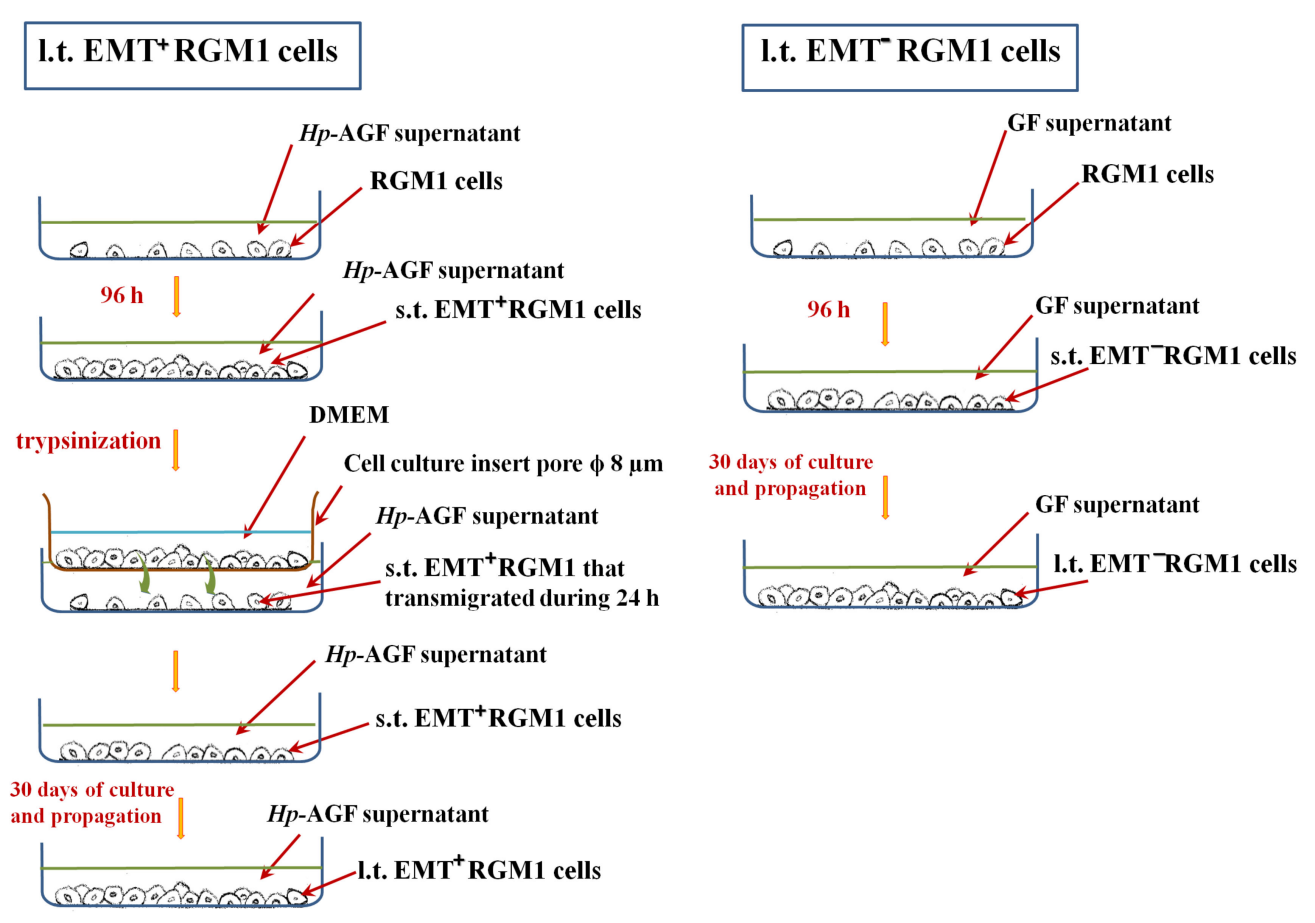
Flowchart of the experimental design.

RGM1 rat gastric epithelial cells [38,39] were cultured in 5 mL of neat *Hp*-AGF supernatant for 96 h on 6-well plates [22,37], then trypsinized (standard trypsinization technique) and resuspended in DMEM with antibiotics and without FBS (Sigma-Aldrich, Saint Louis, MO, USA). Then, the cells were seeded on the upper side of Transwell inserts containing native microporous membranes-pore diameter: 8 μm (Corning, Corning, NY, USA), at a density of 1 × 10^4^ and allowed to transmigrate into the *Hp*-AGF supernatant during 24 h. The most activated post-EMT RGM1 cells which successfully transmigrated through the cell culture inserts were then checked for E- and N-cadherin expression, the indicators of the EMT. E-cadherin negative, N-cadherin positive post-EMT RGM1 cells (abbreviated in our present work as s.t.EMT^+^RGM1 cells) were then cultured in the supernatant from *Hp*-AGFs for at least 30 days (described in our present work as long-term RGM1 cells originating from EMT-positive short-term RGM1 cells l.t.EMT^+^RGM1 cells) in 25 cm^2^ flasks. The neat *Hp*-AGF supernatant was added in an amount of 4 mL and changed every 3 days. Simultaneously, RGM1 cells cultured for 96 h in GF supernatant (abbreviated as s.t.EMT^−^RGM1 cells) were then cultured in GF supernatant for at least 30 days (described in our present study as long-term RGM1 cells originating from short-term EMT negative RGM1 cells (l.t.EMT^−^RGM1 cells). RGM1 cells were cultured in DMEM + 10% FBS (RGM1) for identical period of time (Scheme 1). During all experiments, the amounts of the media as well as the procedures applied to l.t.EMT^+^RGM1 cells, l.t.EMT^−^RGM1, and RGM1 cells were standardized.

The cells were then harvested and used for the mRNA expression analysis by RT-PCR and for protein expression (immunofluorescence and Western blot analysis).

### 2.5. RT-PCR Technique

Total cellular RNA was isolated according to the Chomczynski and Sacchi method [40] using Trizol Reagent (Invitrogen, Carlsbad, CA, USA). First strand cDNA was synthesized from total cellular RNA (2 μg) using Reverse Transcription System (Promega, Madison, WI, USA). Bacterial DNA was isolated from *Hp* strain 43504 using DNAzol Reagent (Life Technologies, Carlsbad, CA, USA) according to the manufacturer’s protocol. The PCR was carried out from l.t.EMT^+^RGM1, l.t.EMT^−^RGM1, and RGM1 cells, using 1 µg cDNA and Promega PCR reagent. Specific primers for *Ki67*, *Cyclin D1*, *Bax*, *Bcl2*, *c-Myc*, *Oct4*, *Sox2*, *Klf4*, *p53*, *TGFβR1*, and *TGFβR2* and *18S* RNA (the verification of the RNA integrity) and 16S bacterial RNA were used (Sigma-Aldrich, Saint Louis, MO, USA). Sequences and annealing temperatures are listed in Table 1. PCR products were separated by electrophoresis in 2% agarose gel containing 0.5 µg/mL ethidium bromide and then visualized under UV light. Location of predicted PCR product was confirmed by using O’Gene Ruler 50 bp DNA ladder (ThermoFisher, Waltham, MA, USA) as standard marker.

### 2.6. Influence of TGFβR1 Kinase Activity Receptor Inhibition on Phenotype of Long-Term RGM1 Cells

Trypsinized l.t.EMT^+^RGM1, l.t.EMT^−^RGM1, and RGM1 cells were seeded at 8 × 10^6^ cells per well and cultured for 96 h in *Hp*-AGF and GF supernatants enriched with TGFβR1 kinase activity inhibitor SB-431542 (10 μM/L) (Sigma-Aldrich, Saint Louis, MO, USA) and in DMEM + 10% FBS, respectively. After incubation period, total cellular RNA was isolated and RT-PCR was carried out (see above). Specific primers for *Ki67*, *Cyclin D1*, *Bax*, *Bcl2*, *c-Myc*, *Oct4*, *Sox2*, *Klf4*, and *18S* RNA (the verification of the RNA integrity) were used (Sigma-Aldrich, Saint Louis, MO，USA) (Table 1).

### 2.7. Cell Proliferation

For proliferation assays, l.t.EMT^+^RGM1, l.t.EMT^−^RGM1, and RGM1 cells were seeded onto 24-well plates at the density of 0.5 × 10^3^ cells/cm^2^ and incubated in 2 mL of *Hp*-AGF supernatant, GF supernatant, and DMEM + 10% FBS, respectively, for 24, 48, 72, and 96 h. Cells were then harvested with trypsin and counted with Bürker’s hemocytometer. The same procedure was applied to l.t.EMT^+^RGM1, l.t.EMT^−^RGM1, and RGM1 cells with the addition of SB-431542 (10 μM/L) (Sigma-Aldrich, Saint Louis, MO, USA). Additionally, the l.t.EMT^+^RGM1 cells were placed in *Hp*-AGF supernatant and in DMEM + 10% FBS and recorded for 12 h using the Leica DMI6000B time-lapse system equipped with a temperature/CO_2_ chamber, the interference modulation contrast optics, and a cooled, digital DFC360FX CCD camera. Then, the number of cell divisions was referred to the total cell count.

### 2.8. Western Blot

Cells were harvested with trypsin and proteins were extracted with the Subcellular Protein Fractionation Kit (Thermo scientific, Waltham, MA, USA). Total protein concentration was determined by nanodrop measurement. Proteins were separated by sodium dodecyl sulfate polyacrylamide gel electrophoresis (SDS-PAGE) (NuPAGE, Invitrogen, Carlsbad, CA, USA) and transferred to nitrocellulose membranes (iBlot System Invitrogen, Carlsbad, CA, USA). Membranes were washed with Tris-buffered saline containing 0.05% Tween-20 (TBST) and blocked with 5% milk in TBST or 3% BSA. Next, the membranes were incubated overnight with primary antibodies: anti-GAPDH, anti-E-cadherin, anti-N-cadherin, anti-LGR5, anti-TGFβR2, and anti-MEK1 at 4 °C and washed with TBST. Then, the membranes were incubated in the presence of HRP-conjugated secondary antibodies for 1 h at RT. The primary antibodies used were: anti-GAPDH (D16H11 Cell Signaling, Merck, Warsaw, Poland), anti-N-cadherin (GTX127345, Genetex, Irvine, CA, USA), anti-E-cadherin (ab212059, Abcam, Cambridge, UK), anti-LGR5 (ab76011, Abcam, Cambridge, UK), anti-TGFβR2 (GTX55814, Genetex, Irvine, CA, USA), and anti-MEK1 (GTX134234, Genetex, Irvine, CA, USA). The HRP-conjugated secondary antibodies used were: goat anti-rabbit IgG (ab6721, Abcam, Cambridge, UK) and goat anti-mouse (ab205719, Abcam Cambridge, UK). Protein detection was carried out with chemiluminescence. Immunoreactive bands were visualized and quantified by imaging densitometry GelPro analyzer (Meyer Instruments, Huston, TX, USA). 

### 2.9. Determination of the Cells Ability to Release TGFβ1

L.t.EMT^+^RGM1, l.t.EMT^−^RGM1, and RGM1 cells were placed on the 6 well plates in DMEM + 10% (0.3 × 10^6^ cells per well) and allowed to release TGFβ for 48 h. Then, the cells supernatants were collected and the concentration of TGFβ1 was measured by ELISA kit no. orb50104 (Biorbyt, St. Louis, MO, USA) according to manufacturer protocol. 

### 2.10. Image Acquisition and Immunofluorescence

Image acquisition was performed with a Leica DMI6000B microscope equipped with the total internal reflection fluorescence (TIRF), Nomarski interference contrast (DIC) modules. LAS-AF deconvolution software was used for image processing. For phenotype plasticity L.t.EMT^+^RGM1, l.t.EMT^−^RGM1 and RGM1 cells were trypsinized and seeded at 8 × 10^6^ cells per well and each of them were cultured for 24 h in all 3 types of media: *Hp*-AGF supernatant, GF supernatant, and DMEM + 10% FBS. Then, the cells were photographed.

For the immunofluorescence, the α-SMA localization was analyzed in formaldehyde (3.7%)-fixed, Triton X-100 (0.1%) permeabilized cells. The mouse anti- α-SMA primary antibody (A2547, Sigma-Aldrich, Saint Louis, MO, USA) was used. Specimens were labeled with Alexa 488-conjugatedgoat anti-mouse IgG (No. A11001, Invitrogen, Carlsbad, CA, USA) and counterstained with Hoechst 33258 (No. B2883, Sigma-Aldrich, St. Louis, MO, USA). For morphological and fluorescent staining evaluation, at least 20 microscopic fields of view were analyzed. 

### 2.11. Statistical Analyses

Statistical analysis of the data was performed with the use of Excel Software. Each variable was expressed as the mean (±S.E.M.). Statistical significance of difference was determined using analysis of variance (one-way ANOVA) test (Statistica Software). Further statistical analysis for post hoc comparisons was carried out with the Newman–Keuls test. Differences were considered statistically at *p* < 0.05.

## 3. Results

### 3.1. Long-Term Influence of Hp-AGF Secretome Induces Phenotypical Changes in Pro-Invasive s.t.EMT^+^RGM1 Cells

**Figure 1:**
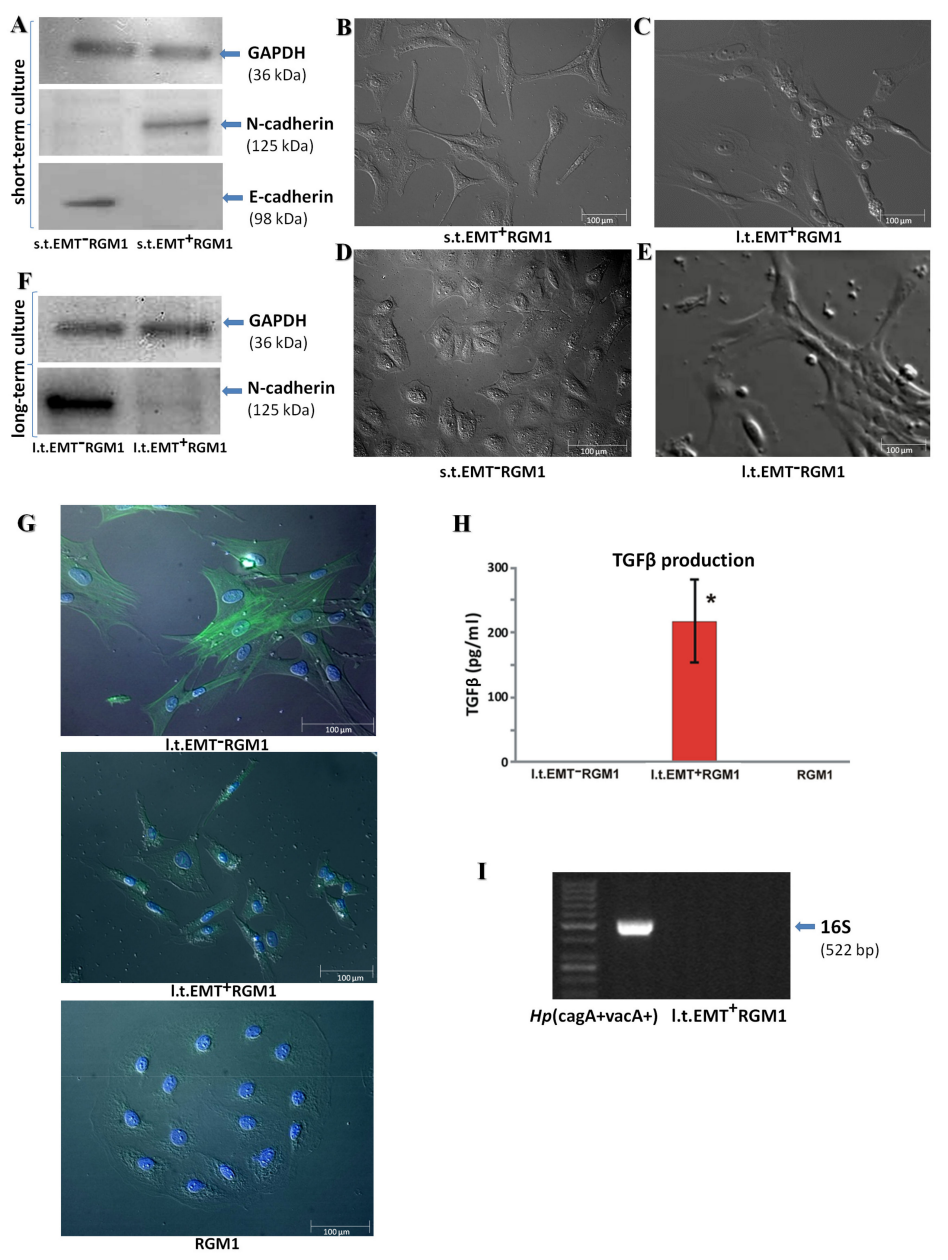
Long-term influence of *Hp*-AGF secretome induces phenotypical changes in pro-invasive EMT^+^RGM1 cells. (**A**) Short-term cell Western blot analysis. Western blot analysis of the EMT process indicators: E- and N-cadherin in total cellular proteins isolated from short-term (96 h) RGM1 cells cultured in gastric fibroblast (GF) secretome (s.t.EMT^−^RGM1 cells) and from short-term (96 h) RGM1 cells cultured in *Hp*-AGF secretome which successfully transmigrated through the Transwell microporous membranes towards *Hp*-AGF secretome during 24 h (s.t.EMT^+^RGM1 cells). The 10 µg of total cellular proteins were loaded per each lane. (**B**) Nomarski contrast of s.t.EMT^+^RGM1; (**C**) l.t.EMT^+^RGM1; (**D**) s.t.EMT^−^RGM1; and (**E**) l.t.EMT^−^RGM1. (**F**) Long-term cell Western blot analysis. Western blot analysis of N-cadherin in total cellular proteins isolated from long-term (30 days) EMT^−^RGM1 cells cultured in GF secretome (l.t.EMT^−^RGM1 cells) and from long-term (30 days) EMT^+^RGM1 cells cultured in the secretome from *Hp*-AGFs (l.t.EMT^+^RGM1), suggesting their reprogramming. The 10 µg of total cellular proteins were loaded per each lane. (**G**) Nomarski contrast and immunofluorescence of α-SMA distribution in original RGM1 cells, l.t.EMT^−^RGM1 and in l.t.EMT^+^RGM1 cells showing differentiation of l.t.EMT^−^RGM1 towards myofibroblasts. Green: α-SMA and blue: chromatin-Hoechst. (**H**) For TGFβ content in the secretome, cells were transferred to DMEM + 10% FBS and allowed for 48 h secretion. The analysis showed that only l.t.EMT^+^RGM1 cells were able to secrete TGFβ. Results are mean ± SEM of six independent experimental repeats. Asterisk indicates a significant change (*p* < 0.05) as compared to the control RGM1 value. (**I**) PCR analysis of the expression of *16S* rRNA showing the absence of *Hp* contamination in l.t.EMT^+^RGM1 cells.

We have previously shown that *Hp* activates paracrine loops between gastric fibroblasts and epithelial cells that result in the phenotypic shifts of RGM1 cells towards rear-front polarized, motile phenotype [14]. To estimate the long-term effects of these loops on the phenotype of RGM1 cells, we allowed these cells to transmigrate through microporous membranes [22]. The cells that most readily (24 h) penetrated the pores were characterized for E- and N-cadherin expression (s.t.EMT^+^RGM1; Figure 1A,B). These cells showed cadherin switch from E- to N-cadherin, which is characteristic for the EMT process. The progeny of these cells was then allowed to expand in the presence of the secretome from *Hp*-AGFs for 30 days (l.t.EMT^+^RGM1; Figure 1C). At the same time, the RGM1 cells were cultured for 96 h in normal gastric fibroblast (GF) supernatant (Figure 1D). In these cells, N-cadherin expression was almost absent, while E-cadherin was expressed (s.t.EMT^−^RGM1; Figure 1A), which remains in agreement with our previous results [22]. Their progeny was allowed to expand in GF supernatant for 30 days. Such long-term incubation in the presence of GF secretome enabled us to establish a fibroblastoid RGM1 lineage (l.t.EMT^−^RGM1; Figure 1E). Western blot analysis revealed the induction of N-cadherin expression in these cells (Figure 1F). Analysis of α-SMA expression revealed that these cells incorporated α-SMA into stress fibers, indicating their myofibroblastic phenotype and GF secretome-induced epithelial-myofibroblast transition (EMyoT; Figure 1G). Concomitantly, an induction of N-cadherin observed after the short-term incubation (s.t.EMT^+^RGM1 cells) in *Hp*-AGF-conditioned medium (Figure 1A) was followed by its down-regulation in the cells that underwent the long-term incubation (l.t.EMT^+^RGM1; Figure 1F). Moreover, α-SMA did not incorporate into stress fibers in l.t.EMT^+^RGM1 cells (Figure 1G). These phenotypic shifts were accompanied by the increased TGFβ1 levels in *Hp*-AGF secretome [22]. Notably, l.t.EMT^+^RGM1 cells were able to release detectable amounts of TGFβ1, as documented for cells allowed to secrete factors in DMEM + 10% FBS for 48 h by ELISA tests (Figure 1H).

### 3.2. The Phenotypic Plasticity of l.t.EMT^+^RGM1 Cells

**Figure 2:**
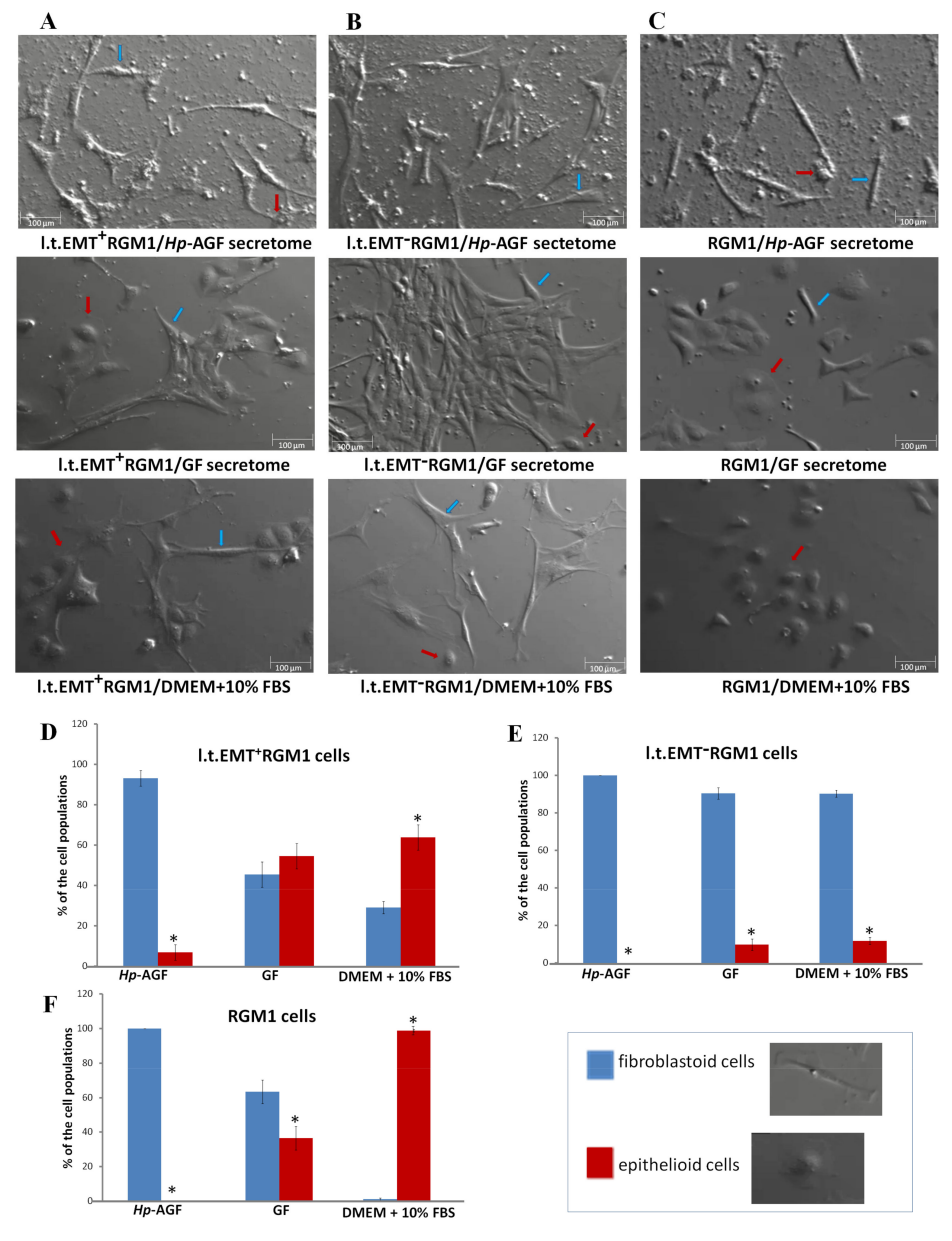
The phenotypical plasticity of l.t.EMT^+^RGM1 cells. Nomarski contrast of the stability of phenotype of (**A**) l.t.EMT^+^RGM1 cells placed in *Hp*-AGF secretome, GF secretome, and DMEM + 10% FBS, respectively; (**B**) l.t.EMT^−^RGM1 cells placed for 24 h in *Hp*-AGF secretome, GF secretome, and DMEM + 10% FBS, respectively; (**C**) and of RGM1 cells placed for 24 h in *Hp*-AGF secretome, GF secretome, and DMEM + 10% FBS, respectively. L.t.EMT^+^RGM1 cells were characterized by plastic phenotype which depended on the type of the medium suggesting EMT/MET switch ability. L.t.EMT^−^RGM1 cells cultured for 24 h in DMEM + 10% FBS possessed stable, fibroblastoid-like phenotype in all tested media. Histogram analysis of morphological cell diversity in (**D**) l.t.EMT^+^RGM1 cells placed in *Hp*-AGF secretome, GF secretome, and DMEM + 10% FBS, respectively; (**E**) l.t.EMT^−^RGM1 cells placed for 24 h in *Hp*-AGF secretome, GF secretome, and DMEM + 10% FBS, respectively; (**F**) and of RGM1 cells placed for 24 h in *Hp*-AGF secretome, GF secretome, and DMEM + 10% FBS, respectively. The cells were counted according to cell morphology (non-polarized epithelioid cells vs rear-front polarized fibroblastoid cells). Blue arrows: sample fibroblastoid cells, red arrows: sample epithelioid cells. Results are mean ± SEM of three to six different independent experimental repeats. Asterisk indicates a significant difference between epithelial and fibroblastoid populations (*p* < 0.05).

L.t.EMT^+^RGM1 cells were characterized by plastic phenotype, which was modified by GF secretome. In the presence of *Hp*-AGF secretome, l.t.EMT^+^RGM1 acquired elongated mesenchymal-like shape with long, thin protrusions (Figure 2A). In the presence of GF secretome, l.t.EMT^+^RGM1 cells retained the elongated, mesenchymal shape with long protrusions or acquired epithelial-like phenotype similar to that of untreated cells (Figure 2A,D). This effect was more pronounced in DMEM + 10% FBS (Figure 2A,D). The cells presented high heterogenicity of morphology with the presence of epithelial-like cells and elongated mesenchymal phenotype with the characteristic polygonal shape, as shown in Figure 2A,D. In turn, l.t.EMT^−^RGM1 cells retained stable fibroblastoid-like morphology in all tested media (Figure 2B,E). Control RGM1 cells had elongated fibroblastoid morphology in the presence of *Hp*-AGF supernatant and epithelial in GF supernatant as well as in DMEM + 10% FBS (Figure 2C,F). 

### 3.3. TGFβR1 Activation Participates in Both Pro-Pluripotent and Pro-Fibrotic RGM1 Reprogramming

**Figure 3:**
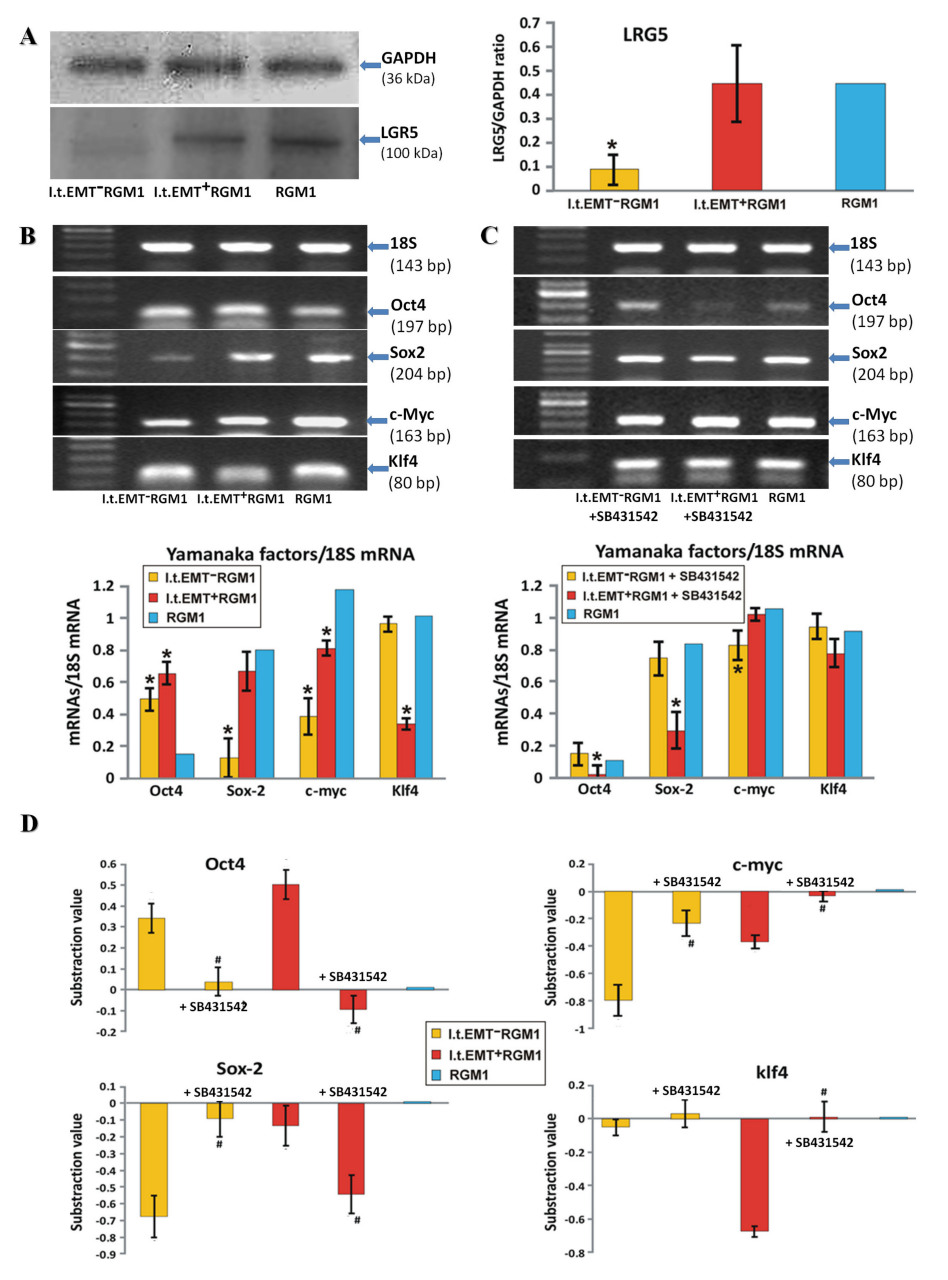
TGFβR1 participates in both pro-pluripotent and pro-fibrotic RGM1 reprogramming. (**A**) Western blot analysis of LGR5 in total cellular proteins isolated from l.t.EMT^−^RGM1 cells, l.t.EMT^+^RGM1 cells, and original RGM1 cells and semi-quantitative densitometry analysis of the ratio of selected proteins over GAPDH. The 10 µg of total cellular proteins were loaded per each lane. (**B**) RT-PCR analysis of the expression of *18S* mRNA and pluripotency indicators Yamanaka factors in l.t.EMT^−^RGM1 cells, l.t.EMT^+^RGM1 cells, and original RGM1 cells and the ratio of selected genes over *18S* mRNA. (**C**) RT-PCR analysis of expression of *18S* mRNA and TGFβR1 signaling dependence of pluripotency indicators Yamanaka factors’ mRNA expression in l.t.EMT^−^RGM1 cells, l.t.EMT^+^RGM1 cells, and original RGM1 cells after addition of TGFβR1 kinase activity receptor inhibitor SB-431542 and the ratio of selected genes over *18S* mRNA. (**D**) The overall analysis of Yamanaka factors’ expression with and without SB-431542 in relation to control value which was taken as zero. The values lower than the controls are represented as negative bars; the values higher than control are represented as positive bars. Results are mean ± SEM of three to six independent experimental repeats. Asterisk indicates a significant change (*p* < 0.05) as compared to the control RGM1 value. Hash indicates a significant change (*p* < 0.05) as compared to the corresponding values without SB-431542.

The presence of TGFβ1 in *Hp*-AGF-conditioned medium prompted us to identify the role of this cytokine in the regulation of *Hp*-AGF-reprogrammed cell plasticity. High levels of LGR5 were retained in l.t.EMT^+^RGM1 cell lineage (Figure 3A). However, they were accompanied by the altered (in comparison to naive RGM1 cells) expression of Yamanaka factors at the mRNA level (Figure 3B). LGR5^+^l.t.EMT^+^RGM1 cells were characterized by the induction of *Oct4* mRNA expression, while it was significantly lower in l.t.EMT^−^RGM1 cells and almost absent in RGM1 cells (Figure 3B) pointing to CSC-like phenotype of l.t.EMT^+^RGM1 cells. Concomitantly, l.t.EMT^+^RGM1 cells retained considerable *Sox2* mRNA expression (the factor responsible for maintaining progenitor cell self-renewal) and slightly lowered, but still pronounced expression of *c-Myc.* Additionally, l.t.EMT^+^RGM1 cells displayed strongly down-regulated *Klf4* encoding mRNA levels (Figure 3B). Thus, l.t.EMT^+^RGM1 cells were characterized by overall LGR5^+^/*Oct4*^high^/*Sox2*^high^/*c-Myc*^high^/*Klf4*^low^ pattern (Figure 3B). The involvement of TGFβ and TGFβR1 activation in the stem-like commitment of l.t.EMT^+^RGM1 cells was further confirmed by the sensitivity of *Oct4*, *Sox2*, *c-Myc*, and *Klf4* to the chemical inhibition of TGFβR1 signaling by SB431542. Consequently, the cells switched to overall *Oct4*^low^/*Sox2*^low^/*c-Myc*^high^/*Klf4*^high^ pattern (Figure 3C,D). On the other hand, LGR5^−^l.t.EMT^−^RGM1 displayed high *Oct4* mRNA expression levels, accompanied by low *Sox2* and *c-Myc* mRNA expression as well as *Klf4* gene up-regulation (overall *Oct4*^+^/*Sox2*^−^/*c-Myc*^−^/*Klf4*^+^ phenotype; Figure 3B). Again, *Oct4*, *Sox2*, *c-Myc*, and *Klf4* mRNA levels were sensitive to TGFβR1 inhibition by SB431542, which resulted in overall *Oct4*^−^/*Sox2*^+^/*c-Myc*^+^/*Klf4*^+^ pattern (Figure 3C,D). Collectively, these data confirm that *Hp*-AGF and naive GF secretome induce alternative reprogramming scenarios, which at least partly depend on TGFβR1 signaling and result in differential LGR5/*Oct4*/*Sox2*/*c-Myc*/*Klf4* expression pattern. 

### 3.4. Hp-AGF Secretome Induces Potentially Pro-Proliferative Phenotype of l.t.EMT^+^RGM1 Cells

**Figure 4:**
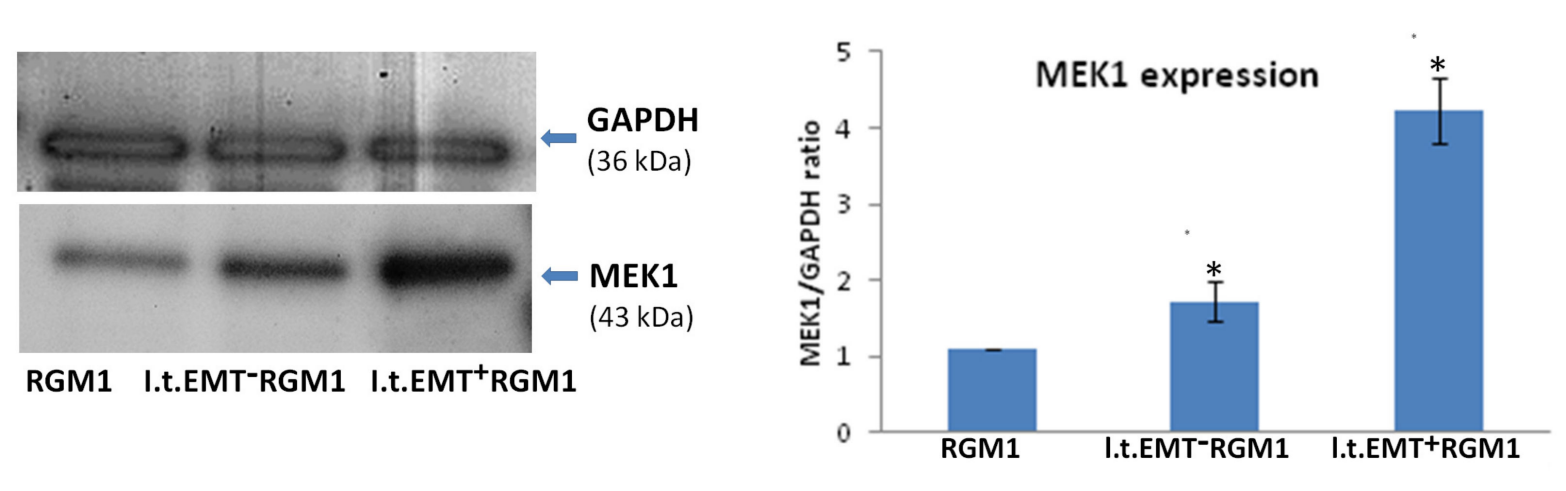
*Hp*-AGF secretome induces potentially pro-proliferative phenotype of l.t.EMT^+^RGM1 cells. Western blot analysis of MEK1 protein expression in total cellular proteins isolated from l.t.EMT^−^RGM1 cells, l.t.EMT^+^RGM1 cells, and original RGM1 cells and semi-quantitative densitometry analysis of the ratio of selected proteins over GAPDH showing the increased expression of MEK1 protein. The 10 µg of total cellular proteins were loaded per each lane. Results are mean ± SEM of three independent experimental repeats. Asterisk indicates a significant change (*p* < 0.05) as compared to the control RGM1 value.

We have checked the pro-proliferative potential of l.t.EMT^+^RGM1 cells cultured in *Hp*-AGF supernatant, l.t.EMT^−^RGM1 cells cultured in GF supernatant, and RGM1 cells cultured in DMEM + 10% FBS by evaluation of MEK1 protein expression. Western blot analysis showed highly accelerated MEK1 expression in l.t.EMT^+^RGM1 cells (Figure 4).

### 3.5. Hp-AGF Secretome Prompts TGFβ-Dependent Proliferation of l.t.EMT^+^RGM1 Cells

**Figure 5:**
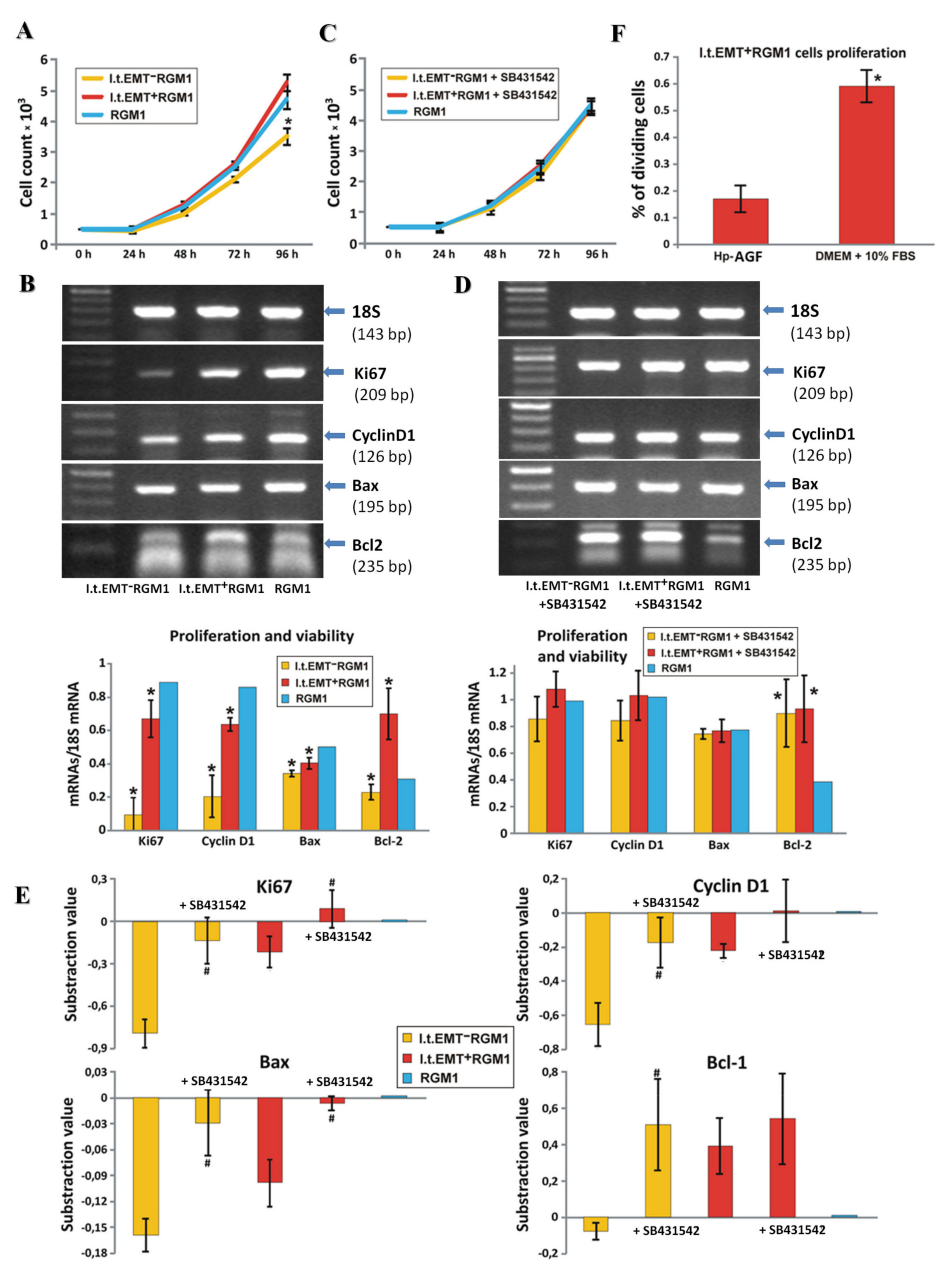
*Hp-AGF* secretome prompts TGFβ-dependent proliferation of l.t.EMT^+^RGM1 cells. (**A**) For the proliferation assay, the l.t.EMT^−^RGM1 cells, l.t.EMT^+^RGM1 cells, and original RGM1 cells were seeded onto 24-well plates (0.5 × 10^3^ cells/cm^2^) in corresponding media. After 24, 48, 72, and 96 h, the cells were harvested, stained with trypan blue, and counted with Bürker’s hemocytometer. (**B**) RT-PCR analysis of the expression of *18S* mRNA and a panel of prosurvival and pro-proliferative factor mRNA expression in l.t.EMT^−^RGM1 cells, l.t.EMT^+^RGM1 cells, and original RGM1 cells and the ratio of selected genes over *18S* mRNA. (**C**) The influence of TGFβR1 signaling on cell proliferation was checked by the addition of 10 µM of TGFβR1 kinase activity receptor inhibitor SB-431542 to the l.t.EMT^−^RGM1 and l.t.EMT^+^RGM1 cells. As a reference, RGM1 cells cultured in DMEM + 10% FBS were used. (**D**) RT-PCR analysis of TGFβR1 signaling dependence of *18S* mRNA and of prosurvival and pro-proliferative factors’ mRNA expression in the presence of SB-431542. (**E**) The overall analysis of proliferative and prosurvival factor expression with and without SB-431542 in relation to the control value which was taken as zero. The values lower than the controls are represented as negative bars; the values higher than control are represented as positive bars (panel **E**). Percentage of dividing l.t.EMT^+^RGM1 cells in *Hp*-CAF secretome and in DMEM + 10% FBS (panel **F**). Results are mean ± SEM of four to six independent experimental repeats. Asterisk indicates a significant change (*p* < 0.05) as compared to the control RGM1 value. Hash indicates a significant change (*p* < 0.05) as compared to the corresponding values without SB-431542.

To substantiate our hypothesis on the involvement of TGFβR1-dependent signaling in the regulation of RGM1 fate by fibroblast secretome, we focused on the proliferation of original l.t.EMT^+^RGM1 cells cultured in *Hp*-AGF supernatant, l.t.EMT^−^RGM1 cells cultured in GF supernatant, and RGM1 cells cultured in DMEM + 10% FBS (Figure 5A). The number of l.t.EMT^+^RGM1 and RGM1 cells was comparable up to 96 h with statistically insignificant, however present increase for l.t.EMT^+^RGM1 at 96 h. In contrast, the number of l.t.EMT^−^RGM1 cells became significantly reduced at 48 h and this inhibitory effect persisted up to 96 h (Figure 5A). 

Differences between the proliferation rate of l.t.EMT^+^RGM1 and l.t.EMT^−^RGM1 populations (Figure 5A) accompanied by their different patterns of pluripotency factors (Figure 3B) and their TGFβR1 dependence (Figure 3C,D) prompted us to estimate the involvement of TGFβR1 in RGM1 growth control. Slight effect of *Hp*-AGF-conditioned medium on *Ki67* and *Cyclin D1* encoding mRNA levels in l.t.EMT^+^RGM1 cells was accompanied by the slight down-regulation of *Bax* and pronounced up-regulation of *Bcl2* in these cells (Figure 5B). Thus, anti-apoptotic effect of TGFβR1, which is present in *Hp*-AGF secretome, may account for the propagation of l.t.EMT^+^RGM1 cells under the selective pressure of *Hp*-AGF secretome. Furthermore, slight inhibition of l.t.EMT^+^RGM1 proliferation (Figure 5C) was accompanied by the up-regulation of *Bax* upon SB431542 administration (Figure 5D,E). Together with the effects on the expression of *Ki67*, *Cyclin D1* mRNA, and less pronounced effect on *Bcl2* mRNA (Figure 5D,E). These findings confirm the important role of TGFβR1 in this process. The percentage of dividing l.t.EMT^+^RGM1 cells allowed proliferating in TGFβ1 rich *Hp*-AGF secretome and in DMEM + 10% FBS for 48 h was significantly higher in DMEM (Figure 5F).

In GF-conditioned medium, l.t.EMT^−^RGM1 cells expressed considerably lower mRNA levels encoding *Ki67* and *Cyclin D1* than their l.t.EMT^+^RGM1 counterparts (Figure 5B), which correlated with their lower proliferation rate (Figure 5A). In contrast to l.t.EMT^+^RGM1 cells, l.t.EMT^−^RGM1 cells reacted to SB-431542 with the induction of proliferation (Figure 5C), correlated with the *Ki67* and *Cyclin D1* gene up-regulation (Figure 5D,E). The expression of *Bax* mRNA underwent slight increase, while the increase of *Bcl2* mRNA was pronounced, which points to the inhibition of apoptosis (Figure 5D,E). Thus, TGFβR1 participates in the induction of anti-apoptotic effect in l.t.EMT^+^RGM1 cells, while it inhibits proliferation and induces apoptosis of l.t.EMT^−^RGM1 cells. Collectively, our data confirm that *Hp*-AGF secretome prompts the enhanced proliferation of l.t.EMT^+^RGM1 under TGFβ1 stress, thus potentially facilitating *Hp*-induced gastric neoplasia. 

### 3.6. Interrelations between TGFβR1 and TGFβR2 Activity Underlie Differential Microevolution Pattern of l.t.EMT^+^RGM1 and l.t.EMT^−^RGM1 Cells

**Figure 6:**
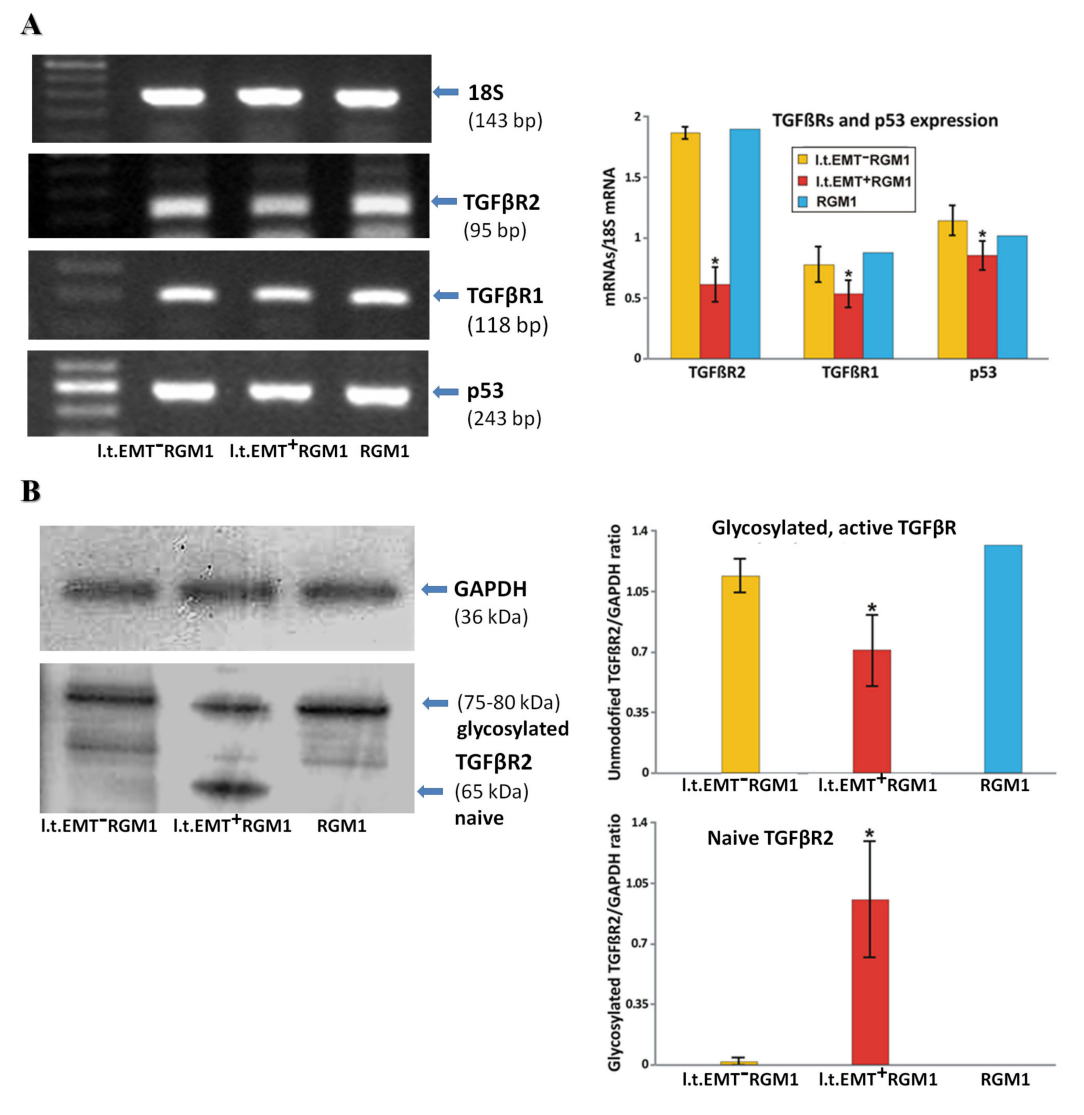
Differential microevolution pattern of RGM1 cells is associated with differently expressed TGFβRs. (**A**) RT-PCR analysis of the expression of *18S* mRNA and of *TGFβRs* and *p53* mRNA in l.t.EMT^−^RGM1 cells, l.t.EMT^+^RGM1 cells, and original RGM1 cells and the ratio of selected genes over *18S* mRNA showing differentially expressed *TGFβRs* mRNA and decreased mRNA expression of the tumor suppressor *p53* in l.t.EMT^+^RGM1 cells. (**B**) Western blot analysis of TGFβR2 protein expression in total cellular proteins isolated from l.t.EMT^−^RGM1 cells, l.t.EMT^+^RGM1 cells, and original RGM1 cells and semi-quantitative densitometry analysis of the ratio of selected proteins over GAPDH, showing the presence of two forms of TGFβR2: naïve and active, glycosylated form of TGFβR2 in l.t.EMT^+^RGM1 cells. The 10 µg of total cellular proteins were loaded per each lane. Results are mean ± SEM of four to six independent experimental repeats. Asterisk indicates a significant change (*p* < 0.05) as compared to the control RGM1 value.

Apparently, secretome of gastric fibroblasts can switch between alternative developmental programs in RGM1 cells, depending on gastric fibroblast activation by *Hp*. Further experiments were performed to address the TGFβ-related mechanisms underlying differential reactivity of l.t.EMT^+^RGM1 and l.t.EMT^−^RGM1 cells to *Hp*-AGF and GF secretomes. Even though *TGFβR1* mRNA levels were slightly down-regulated in l.t.EMT^+^RGM1 cells, *TGFβR1* and *TGFβR2* genes were activated in all tested cell types, both in the presence/absence of *Hp*-AGF secretome (Figure 6A). A more pronounced down-regulation of *TGFβR2* mRNA expression in l.t.EMT^+^RGM1 cells (Figure 6A) was accompanied by the presence of two bands in Western blot analysis (Figure 6B). The lower band (ca. 65 kDa) had the molecular mass of unmodified TGFβR2 protein, whereas the second (75–80 kDa) corresponded to the glycosylated (active) TGFβR2 [41] (Figure 6B). These data point to partial inactivation of TGFβR2 in l.t.EMT^+^RGM1 cells as indicated by decreased levels of glycosylated TGFβR2 compared to l.t.EMT^−^RGM1 and RGM1 cells (Figure 6B). l.t.EMT^+^RGM1 cells were also characterized by decreased *p53* mRNA level (Figure 6B).

The data indicate that the collective TGFβR1/2 activation determines the expansion of pro-invasive RGM1 cells. Apparently, remaining TGFβR1 activity along with TGFβR2 stimulation may negatively affect the proliferation of l.t.EMT^−^RGM1 cells in the presence of GFs secretome. This mechanism is potentially involved in the regulation of l.t.EMT^−^RGM1 cell stromal phenotype. In turn, TGFβ1 can induce pro-neoplastic expansion of l.t.EMT^+^RGM1 cells under conditions of the reduced TGFβR2 signaling. These findings underline the importance of TGFβ signaling balance for the regulation of phenotypic fate of gastric epithelial cells.

## 4. Discussion

Tumors comprise heterogeneous cell populations with distinct phenotypes, functions, and gene expression profiles [42]. Currently, a growing body of evidence supports the view that gastric cancer is initiated by single self-renewing CSC-like cells [30,43,44]. CSCs have the potential to self-renew and differentiate, thus initiating and sustaining the primary tumor growth and establishing metastases [33,45,46]. However, CSC origins still remain a matter of debate. During tumor development, CSCs cooperate with stromal fibroblasts that constitute the milieu for neoplastic loci and the barriers for immune system, concomitantly providing paracrine signals for tumor promotion [18,26,27,28,29]. We have previously reported that normal gastric epithelial RGM1 cells undergo EMT-like phenotypic shifts, accompanied by inhibition of proliferation upon short-term (96 h) exposure to the secretome from *Hp*-infected gastric fibroblasts (*Hp*-AGFs) [22]. Here, we demonstrate that the prolonged cultivation of these post-EMT RGM1 cells in the presence of *Hp*-AGF secretome induces their reprogramming towards overall LGR5/*Oct4* high phenotype, characteristic for phenotypically plastic stem-like neoplastic cells. In turn, the induction of RGM1 reprogramming towards the stromal α-SMA+ phenotype was seen in the presence of non-infected gastric fibroblast (GF) secretome, confirming the role of *Hp* in biasing the GF secretome towards pro-EMT activity. Apparently, TGFβR1/TGFβR2-dependent signaling switches participate in these alternative scenarios. Thus, paracrine loops within pre-cancerous loci govern the developmental fate of gastric epithelium via directing epithelial cells towards neoplastic or stromal differentiation program in aTGFβR1/R2-dependent/*Hp* regulated manner.

Apparently, "trans differentiation" processes and the induction of pluripotency factors promote the phenotypic plasticity of the cells within pre-neoplastic loci that facilitates the adaptation of alternative differentiation programs by gastric epithelial cells. We show that RGM1 cells can undergo alternative reprogramming events, depending on the *Hp*-modulated quality of humoral factors secreted by gastric fibroblasts. In the long-term presence of *Hp*-AGF secretome, RGM1 cells evolve towards the plastic *Oct4^+^*/LGR5^+^ phenotype (l.t.EMT^+^RGM1). *Oct4* stands at the top of a hierarchy governing the regulation of both pluripotency and de-differentiation [30,47,48,49]. In addition, LGR5^+^ GC cells have previously been shown to display stem cell-like features [50,51] and to regulate Oct4 levels. The overall LGR5/*Oct4* expression in phenotypically plastic l.t.EMT^+^RGM1 cells suggests their stem-cell like pluripotency commitment, which is potentially relevant for tumor development [30,52,53]. Thus, *Hp* induces fibroblast activation towards CAFs [35], which secrete factors increasing LGR5/*Oct4*-dependent stem cell-like potential of epithelial RGM1 cells, while non-infected fibroblasts are more predisposed to direct gastric epithelium towards the stromal (pro-fibrotic) phenotype. Such microevolution of fibroblastoid cells with overall *Oct4^+^*/LGR5^−^ phenotype was observed in RGM1 populations exposed for long periods to the GF secretome. l.t.EMT^−^RGM1 EMyoT-related phenotypic commitment is illustrated by α-SMA incorporation into stress fibers, l.t.EMT^−^RGM1 relative dormancy and stable (niche-secretome independent) myofibroblastoid phenotype.

Induction of complementary developmental scenarios in gastric epithelium may have a profound significance for *Hp*-induced initiation of gastric tumorogenesis. Nevertheless, further studies are necessary to address the multipotency and invasiveness of l.t.EMT^+^RGM1 cells, their *Oct4*^+^/LGR5^+^ expression accompanied by transient N-cadherin up-regulation, the post-EMT morphology/motility and apparent phenotypic plasticity suggest their CSC-like properties. Thus, we show the interrelations between mesenchymal phenotype and plasticity of cancer cells in the in situ GC model. Incomplete, partial EMT has previously been shown to account for the phenotypic plasticity (the ability to produce more than one phenotype) of cancer cells [24,30,33,54,55,56,57,58,59]. These interrelations also participate in the EMT/MET-dependent GC progression, where epithelioid cancer cells undergo EMT to enter the metastatic cascade, whereas post-EMT CSCs undergo MET-directed differentiation after metastatic niche nesting [24,30,60,61]. Gastrointestinal CSCs were shown to have elevated expression levels of genes associated with stemness and pluripotency. Apart from Lgr5, Oct4, these include c-Myc, Sox2, and Klf4 [30,62,63,64,65,66] discrete overall expression patterns of these factors in l.t.EMT^+^RGM1 and l.t.EMT^−^RGM1 cells confirm their involvement in the alternative EMT/EMyoT-related differentiation scenarios induced in gastric epithelial cells by their paracrine looping with *Hp*-AGFs/GFs, respectively. Apparently, prominent *Klf4* and less pronounced *c-Myc* down-regulation facilitates pre-neoplastic phenotype of otherwise *Oct4*^+^/LGR5^+^ l.t.EMT^+^RGM1 cells, whereas LGR5/*Sox2*/*c-Myc* overall down-regulation is linked to pro-fibrotic/stromal phenotype of l.t.EMT^−^RGM1 cells. Consequently, these key modulators of pluripotency [33,67,68,69] are closely linked not only to *Hp*-AGF-induced EMT of gastric epithelial cells but also participate in their non-infected fibroblast-induced EMyoT.

It has also been stated, that MEK1 and MEK2, which are related protein kinases involved in the Ras/Raf/MEK/ERK signal transduction cascade, participate in the regulation of cell cycle progression, cell differentiation, and proliferation [70], which suggests that the effect of the supernatant on proliferation rate could not account for the nutrient depletion by fibroblasts. The Ras-dependent ERK1/2 MAP kinase signaling pathway plays a central role in cell proliferation control and is frequently activated in different human cancers. Early studies have shown that expression of activated alleles of MEK1 is sufficient to deregulate the proliferation and trigger the morphological transformation of cell lines [70,71,72,73,74]. Moreover, ERKs have been shown to be vital for CSC tumorogenicity [75]. 

Previously, short-time (96 h) incubation of RGM1 cells in the presence of *Hp*-AGF secretome has been shown to induce *TGFβR1*/*R2* mRNA up-regulation in these cells (especially *TGFβR2*) [37]. It is well established that TGFβ1 induces EMT during pathologic and physiologic fibrosis of normal tissues [22,23,24,25,37,76,77,78,79,80,81,82]. Pleiotropic effects of TGFβ1 are also observed in cancer systems, where TGFβ signaling can suppress or promote tumor initiation [80,81,82] and progression [83,84,85]. Relatively high *Lgr5*/*Oct4*/*Sox2* mRNA levels, accompanied by *p53* down-regulation [86,87], high proliferation rate, and TGFβ1 secretion by l.t.EMT^+^RGM1 cells indicate that TGFβ1 is involved in the induction of their CSC-like/proliferative state. The *p53* down-regulation in l.t.EMT^+^RGM1 cells may sustain the activity of TGFβ1 signaling in l.t.EMT^+^RGM1 cells at the levels promoting their viability. Furthermore, *Klf4* down-regulation in l.t.EMT^+^RGM1 cells remains in concordance with this concept, as Klf4 promotes differentiation through TGFβ1 target gene activation and myofibroblast formation [88]. In a parallel manner, Klf4 suppresses oncogenic TGFβ1 signaling [89] via interactions with TGFβ1 control elements (TCEs) [90]. TGFβ1 is considered an immune biomarker which has also been linked to myocardial function and remodeling due to the regulation of valve fibrosis and calcification processes [91]. Notably, Klf4 has been shown to be down-regulated during tumor initiation and consecutively lost during GC progression [92,93,94]. Furthermore, the involvement of TGFβ1 in the regulation of pro-neoplastic l.t.EMT^+^RGM1 phenotype is evidenced by the shifts in overall *Oct4*/*c-Myc*/*Sox2*/*Klf4* mRNA levels following TGFβR1 inhibition. Additionally, anti-apoptotic activity of TGFβR1 is evidenced by some slight inhibition of l.t.EMT^+^RGM1 cell proliferation and *Bax* up-regulation induced by SB-431542. Thus, *Hp*-AGFs help to sustain the EMT-related CSC commitment of RGM1 cells and their viability in a TGFβ1-mediated, TGFβR1-dependent, and *Hp*-modulated manner. This hypothesis was further confirmed by relatively strong impact of TGFβR1 inhibition on *Oct4*/*c-Myc* and *Sox2* expression pattern in l.t.EMT^−^RGM1 cells. This finding indicates that TGFβR1 signaling remains active in these cells even in the environment characterized by lower level of TGFβ1 [22] and is necessary to sustain their myofibroblastic phenotype. This observation was accompanied by SB431542-induced l.t.EMT^−^RGM1 proliferation, which was correlated with *Ki67*, *Cyclin D1*, and *Bax* mRNA up-regulation back to the levels characteristic for naive RGM1 cells. The suppression of nuclear translocation of β-catenin into gastric cells along with the expression of the β-catenin target survival genes *c-myc* and *cyclin D1* along with induction of apoptosis has recently been implicated as a potential mechanism of anti-cancer therapy [95]. Collectively, these observations confirm that non-infected GF secretome contains the range of TGFβR1 ligands that determine the array of developmental fates undertaken by RGM1 cells [96]. In conjunction with apparently opposite (inducing) effects of TGFβ1/TGFβR1 on the phenotype/proliferation of l.t.EMT^+^RGM1 cells, this observation also indicates that these ligands cooperate with TGFβ1 in biasing RGM reprogramming towards pro-neoplastic phenotype, while prompting EMyoT in the lower concentrations of TGFβ1. Relatively low TGFβR2 expression/activity and *p53* impairment [87,97,98] in l.t.EMT^+^RGM1 cells may suggest the role of TGFβR1/R2 interactions in the differential effects of *Hp*-AGF/GF secretome on RGM1 phenotype. Accordingly, the combination of TGFβ1 with the other TGFβR influencing ligands impairs TGFβR2 activity, prompting the neoplastic program in RGM1 cells, whereas their myofibroblastic differentiation is induced in the low level of TGFβ1/presence of active TGFβR2. These findings need further research comprising direct and indirect influence of *Hp* on reciprocal interactions between different cellular components of gastric tissue taking place in vivo.

## 5. Conclusions

Collectively, our data confirm the primary role of the signals from *Helicobacter pylori* in the remodeling of gastric niche towards pro-neoplastic activity. In the absence of *Hp*, occasional lesions can temporarily help to establish potential paracrine loops between gastric stromal fibroblasts and epithelial cells, induce EMyoT in gastric epithelium, and prompt local tissue regeneration. On the perimeter of chronic *Hp*-induced lesions, where gastric fibroblasts are activated by local inflammation but not directly exposed to *Hp* signals, EMyoT of gastric epithelium can turn into the chronic state. On the other hand, direct *Hp*-generated stress results in further reprogramming of gastric fibroblasts and stabilization of paracrine loops between *Hp*-infected stromal fibroblasts possessing CAF-like characteristic [35] and gastric epithelium. *Hp*-infected stromal fibroblasts prompt reprogramming of normal gastric epithelial cells towards precancerous phenotype. These events are related to TGFβ1 release by *Hp*-AGF/epithelium, which modulates TGFβR1/R2-dependent signaling, induces EMT in non-cancerous epithelial cells, consequently prompting their pro-CSC-like phenotype. Consequently, the post-EMT cells are reprogrammed towards TGFβ1-dependent expansion and CSC-like commitment, providing the basis for their further EMT/MET plasticity. The *Hp*-activated fibroblast reprogramming of epithelial cells may thus potentiate the canonical pathway of *Hp* infection influence on epithelial cells.

## Figures and Tables

**Table 1:** Rat oligonucleotide primers for detection of mRNA and *Hp* oligonucleotide primer for detection of *16S* rRNA by RT-PCR and PCR, annealing temperature and size of PCR products employed in the experimental protocol.

Gene	Primer Sequence	Size of PCR Product (bp)	Annealing Temp. (°C)
***18S***	Forward 5′-GTTGGTTTTGATCTGATAAATGC-3′ Reverse 5′-CATTAAATCAGTTATGGTTCCTTTG-3′	**143**	**60**
***Bax***	Forward 5′-CTGCCAACCCACCCTGGTCT-3′ Reverse 5′-TGGCAGCTGACATGTTTTCTG-3′	**195**	**55**
***BCL2***	Forward 5′-ACTGAGTACCTGAACCGGCATC-3′ Reverse 5′-GGAGAAATCAAACAGAGGTCGC-3′	**108**	**60**
***c-Myc***	Forward 5′-CCACACAGCCCACTGGTCCT-3′ Reverse 5′-GGCTGGAGCATTTGCGGTTGTT-3′	**163**	**60**
***Cyclin D1***	Forward 5′-TGCTTGGGAAGTTGTGTTGG-3′ Reverse 5′-AATGCCATCACGGTCCCTAC-3′	**126**	**60**
***c-Myc***	Forward 5′-CCACACAGCCCACTGGTCCT-3′ Reverse 5′-GGCTGGAGCATTTGCGGTTGTT-3′	**163**	**60**
***Ki-67***	Forward 5′-AACCAGGACTTTGTGCTCTGTAA-3′ Reverse 5′-CTCTTTTGGCTTCCATTTCTTC-3′	**209**	**60**
***Klf4***	Forward 5′-TTCTCCACGTTCGCGTCCGG-3′ Reverse 5′-TCTCGCCAACGGTTAGTCGGGG-3′	**80**	**60**
***Oct4***	Forward 5′-GGAGGGATGGCATACTGTGGACCT-3′ Reverse 5′-TCCTGGGACTCCTCGGGACTAGG-3′	**197**	**60**
***p53***	Forward 5′-AGTGAAGGGACTAGCATTGTC-3′ Reverse 5′-GGATGCCCGTGCTGCCGAGGAG-3′	**243**	**60**
***Sox2***	Forward 5′-AGAACCCCAAGATGCACAAC-3′ Reverse 5′-CTCCGGGAAGCGTGTACTTA-3′	**204**	**60**
***TGFβ rec 2***	Forward 5′-TGTGGCAGAGCGCTTCAGT-3′ Reverse 5′-TGTTCAGGGAGCCGTCTTCT-3′	**95**	**60**
***TGFβ rec 1***	Forward 5′-GCAGACTGGACCAGCAATGAC-3′ Reverse 5′-CTGCAATCAGGATCACTGCAA-3′	**118**	**60**
***Hp 16S***	Forward 5′-GTCAAGAGATCAGCCTATGTCC-3′ Reverse 5′-TGGCAATCAGCGTCAGGTAATG-3′	**522**	**54**
